# Inheritance Pattern of Temephos Resistance, an Organophosphate Insecticide, in *Aedes aegypti* (L.)

**DOI:** 10.1155/2015/181872

**Published:** 2015-03-16

**Authors:** Vinaya Shetty, Deepak Sanil, N. J. Shetty

**Affiliations:** Centre for Applied Genetics, Bangalore University, Jnana Bharathi Campus, Bengaluru 560056, India

## Abstract

The present paper reports the mode of inheritance of resistance in laboratory induced temephos resistant and susceptible strains of *Ae. aegypti*. Homozygous resistant and susceptible strains of *Ae. aegypti* were generated by selective inbreeding at a diagnostic dose of 0.02 mg/L of temephos. Genetic crosses were carried out between these strains to determine the inheritance pattern of temephos resistance. The log-dosage probit mortality relationships and degree of dominance (*D*) were calculated. The dosage-mortality (*d*-*m*) line of the *F*_1_ generation was nearer to the resistant parent than the susceptible one. The “*D*” value was calculated as 0.15 indicating that the temephos resistant gene is incompletely dominant. The *d*-*m* lines of the *F*_2_ generation and progeny from the backcross exhibited clear plateaus of mortality across a range of doses indicating that temephos resistance is controlled by a single gene. Comparison of the mortality data with the theoretical expectations using the *χ* ^2^ test revealed no significant difference, confirming a monogenic pattern of inheritance. In conclusion, the study provides evidence that the temephos resistance in *Ae. aegypti* follows an incompletely dominant and monogenic mode of inheritance.

## 1. Introduction

*Aedes aegypti* (Diptera : Culicidae), the principal vector of dengue fever (DF) and its more severe form dengue hemorrhagic fever (DHF), is of significant public health concern in tropical, subtropical, and temperate regions of the world [1, 2]. The global prevalence of the disease has grown dramatically in recent decades. World Health Organization (WHO) estimates about two-fifths of the world's populations at risk of dengue infection [3]. Vector control has been a significant strategy to control mosquito-borne diseases worldwide. Currently, chemical treatment is the most important component in an integrated campaign to achieve effective vector control [4]. The repeated use of insecticides to control the mosquito population is believed to be the main source of resistance in these vectors [5, 6] and is considered as a recent evolutionary adaptation to changes in the environment, arising in less than a century, in response to the repetitive use of chemical insecticides [7].

*Ae. aegypti* is an extremely successful species when it comes to the ability to disperse and to adapt to varying environments. Currently the most feasible, effective, and practical method of control of this vector species is through the use of insecticides [8]. Temephos, an organophosphate (OP) insecticide, is recommended as a larvicide by World Health Organization (WHO) to control mosquitoes, midge, blackfly, and other insects [2]. In India, it has been recommended for controlling larval stages of mosquitoes and its use is patronized by the Government of India under their National Vector Borne Diseases Control Programme (NVBDCP), Defense Services, and so forth. It is also used in several other countries like Brazil, USA, South Africa, and Southeast Asian countries for mosquito control programme [2, 9]. Temephos is widely used for the control of* Aedes*. However, its indiscriminate use has led to development of resistance against temephos, in Cambodia, federal districts of Brazil, and Southeast Asia [9–14]. Studies have shown temephos to be effective in controlling* Ae. aegypti* in several parts of India [15, 16]. However, different populations of Bangalore and Mumbai have shown a high tolerance to temephos, when compared with the WHO recommended diagnostic dose [17, 18]. Inherited resistance to some of the insecticides such as DDT and Dieldrin in* Ae. aegypti* has been identified and reported [19, 20]. Understanding the mode of inheritance helps in resistance detection, monitoring, modeling, and risk assessment [21]. Hence, the present study is of considerable significance in deciphering the inheritance pattern of temephos resistance in the said species.

Resistance mechanism such as increased sequestration or detoxification causes a reduced dose of the insecticide at target site, while reduced target site sensitivity causes ineffective binding of the insecticide at a given dose [22–24]. Acquisitions of resistance to insecticides can be used to gauge the microevolution process, because, as a result of this compelling selection pressure, the rate of evolution is higher [25]. Considering that such acquired resistance is the reflection of changes in the genotypic architecture of natural populations, investigating the genetic basis of resistance is a prerequisite to understand the evolution of this phenomenon [26]. Besides the evolutionary approach, there is also an urgent need to improve our knowledge of the mechanisms governing resistance development. The present paper describes the mode of inheritance in laboratory induced temephos resistant and temephos susceptible strains in* Ae. aegypti*.

## 2. Materials and Methods

### 2.1. Mosquito Rearing

*Aedes aegypti* used in the present study was originally collected from Jaya Prakashnarayan Nagar (JPN), Bangalore, India. The larvae and adults were reared in an insectary maintained at 25 ± 1°C, relative humidity 75 ± 5%, and a 14-hour photoperiod [27]. Adults were maintained in cages of iron frames covered by cotton net cloth and fed on 10% sucrose solution in a jar with a cotton wick. Polypropylene cups (3′′ diameter) lined with filter paper and containing clean tap water were placed inside the cages for oviposition. Powdered mixture of yeast tablets (Geo Pharmaceuticals, Bangalore) and dog biscuits (Pedigree, Mars Industries, Hyderabad) were provided as larval diet. All mosquitoes used in the experiments were reared at a density of around 600 larvae per tray (25 × 30 cm) containing approximately 2 litres of water (water depth 2 cm).

### 2.2. Insecticide

Temephos (Abate) (50% TC), an organophosphate insecticide, with molecular formula C_16_H_20_O_6_P_2_S_3_, and International Union for Pure and Applied Chemistry (IUPAC) name *O*, *O*, *O*′, *O*′-tetramethyl *O*, *O*′-thiodi-*p*-phenylene bis(phosphorothioate), was used in the present study.

### 2.3. Larval Bioassay

Denatured alcohol (98 mL of absolute alcohol and 2 mL of methyl ethyl ketone) was used as the solvent to prepare a range of stock concentrations of temephos [28, 29]. The initial phase of the study involved exposing 25 early fourth instar larvae contained in 500 mL glass beakers with 1 mL of the designated concentration of insecticide and made up to 250 mL with dechlorinated tap water. A range of increasing concentrations was used to get the mortality between 2% and 98% after a 24 h exposure. Each concentration had four replicates. Controls were set up identically using 1 mL of ethanol to 249 mL of tap water, without the insecticide. Mortality in each case was recorded after 24 hrs of exposure to the insecticide and the mortality percentage was calculated by including both dead and moribund larvae as per WHO guidelines [28, 29].

### 2.4. Development of Temephos Resistant and Susceptible Strains

*Temephos Resistant (TR) Strain.* According to the procedure of WHO, susceptibility studies were carried out for the late third instar larvae from the isofemale lines of JPN strain with WHO diagnostic dose of 0.02 mg/L [28, 29]. Twenty-four hours later, the surviving larvae from the test showing lowest mortality of isofemale population were collected, maintained separately, and used for inbreeding. Mass treatment was followed to treat the larvae of successive generations, and the surviving ones were inbred to obtain further generations. The process of selective inbreeding was repeated by gradually increasing the dose from subdiagnostic concentrations to 0.02 mg/L until a pure homozygous resistant (100% survival) strain was established.

*Temephos Susceptible (TS) Strain.* JPN strain was used to select homozygous susceptible strain. About 50% of the larvae obtained from the isofemales of JPN strain were treated to the diagnostic dose of 0.02 mg/L. Untreated larvae of the batch showing the highest percentage of mortality were selected for inbreeding and the selection procedure was repeated until getting a pure homozygous susceptible strain (100% mortality).

### 2.5. Genetic Studies of TR

Twenty-five pairs of freshly emerged males and females of the homozygous resistant (*R*) and susceptible (*S*) strains were used, to carry out reciprocal genetic crosses (*R*♂ × *S*♀ and *R*♀ × *S*♂). A part of the *F*_1_ individuals was inbred to get *F*_2_ generation and the remaining mosquitoes were backcrossed (*F*_1_ × *S*) by reciprocal cross of both male and female progeny to parental type (*S*). Apart from this, the late third instar larvae from all the crosses were subjected to larval bioassays. The log-dosage probit mortality relationships were recorded for all the genetic crosses [30–33] and the degree of dominance (*D*) was calculated using Stone's formula [34]:

(1) $$\begin{array}{c} D=\frac{2X_{2}-X_{1}-X_{3}}{X_{1}-X_{3}}, \end{array}$$

where *D* is the degree of dominance and *X*_1_, *X*_2_, and *X*_3_ are the logarithms of the LC_50_ (concentration that produces 50% mortality) values of the resistant, *F*_1_ hybrid, and susceptible strains, respectively.

The value calculated for *D* indicates whether the trait is completely dominant (*D* = 1), incompletely dominant (0 < *D* < 1), incompletely recessive (−1 < *D* < 0), or completely recessive (*D* = −1).

### 2.6. Data Analysis

The LC_50_ and LC_90_ values of the bioassay were calculated by subjecting the log-dosage-mortality data to probit analysis [31]. The dosage-mortality lines (*d*-*m* lines) which give an insight into the mode of inheritance were constructed. Microsoft Office Excel 2007 was employed for data analysis. Controls were set up to determine natural mortality and Abbot's formula was used to correct the mortality data from larvicidal assays [35]. Chi square (*χ* ^2^) values were calculated using the procedure of Bailey [36].

## 3. Results

**Figure 1:**
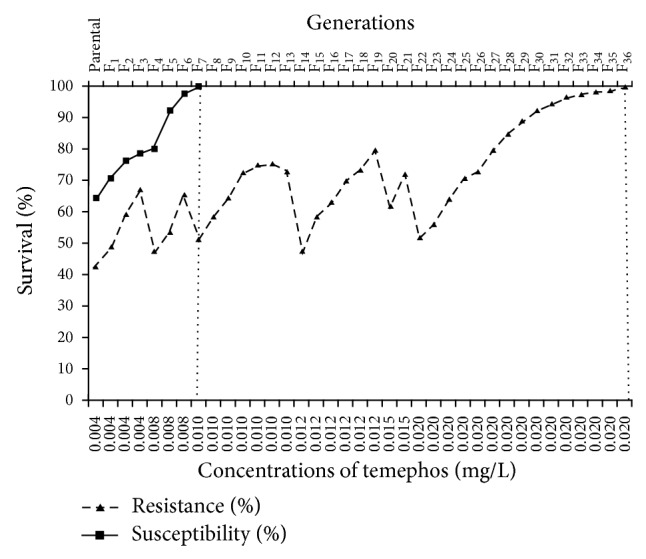
Development of homozygous resistance and susceptible strains of* Aedes aegypti* in each generation. The line showing the 100% susceptibility and resistance after 7 and 36 generations at 0.01 and 0.02 mg/L, respectively.

**Figure 2:**
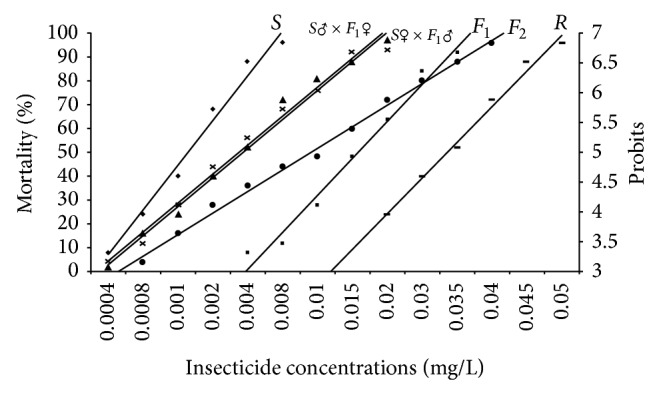
Dosage-mortality relationships of the temephos resistance and susceptible strains of* Aedes aegypti*. The dosage-mortality lines were constructed for the larvae from all the crosses including parental (*S*, *R*), reciprocal (*F*_1_), and backcrosses (*S*♂ × *F*_1_♀, *S*♀ × *F*_1_♂) and also for *F*_2_ generation.

Homozygous resistant and susceptible strains of* Ae. aegypti* were synthesized in laboratory by continuous selection and inbreeding for 36 and 7 generations, respectively, using 0.02 mg/L diagnostic dose of temephos (Figure 1). Results of these crosses are presented in Table 1. Crosses 1 and 2 established homozygous resistant and susceptible strains, showing clear homozygosity to resistance (100% survival) and susceptibility (100% mortality), respectively. The LC_50_ of resistant strain was 56.25 times greater than that of susceptible strain. Bioassay of the parental strains with temephos yielded a straight *d*-*m* line, indicating the purity of gamete for resistance and susceptibility (Figure 2). Reciprocal crosses (crosses 3 and 4) between the resistant and susceptible strains resulted in *F*_1_ hybrids which exhibit 56.88% and 59.75% of resistance, respectively. Backcrossing the *F*_1_ hybrids from crosses 3 and 4, to their homozygous parent line, yielded progeny with 54.93%, 57.04%, 56.96%, and 53.95% resistance, respectively (crosses 5, 6, 7, and 8). *F*_2_ progeny from crosses 9 and 10 showed 55.44% and 59.71% resistance, respectively, using the data derived from these crosses, and the log *d*-*m* lines (Figure 2) were constructed. The *d*-*m* lines of backcrosses were found to be in between susceptible and *F*_1_ hybrids (Figure 2). As observed in Figure 2, the *d*-*m* line of *F*_1_ was inclined towards the resistant line and “*D*” value was calculated to be 0.15. Using Chi square test, the mortality data of the progeny from the backcrosses were compared with theoretical expectations and tested for monogenic inheritance. Incomplete dominance was apparent in the progeny of crosses 3 and 4, considering that they exhibited slightly over 50% resistance and the fact that the position of the log dose probit line was towards the resistant parent.

The expected segregation of the backcross of the *RS* (heterozygous) to the *S* (homozygous susceptible) strain for monogenic Mendelian inheritance was calculated using the formula [37]

(2) $$\begin{array}{c} x\left(BC\right)=\left(\frac{1}{2}\right)a_{1}\left(RS\right)+\left(\frac{1}{2}\right)a_{1}\left(S\right), \end{array}$$

where *x* is expected response of the backcross at a particular dose and *a*_1_ and *a*_2_ are observed responses of *RS* and *S* populations at that dose.

In this instance, at the diagnostic dose, a 50% survival is expected in the backcrosses, since the mortality of all susceptible individuals (*S*) would leave behind 50% heterozygotes in the progeny. Accordingly, 50% survival is observed in the crosses 5, 6, 7, and 8 with slight deviations which are nonsignificant at *P* < 0.05. Applying the same principle, the expected *F*_2_ segregation was calculated using formula [37]

(3) $$\begin{array}{c} x\left(F_{2}\right)=\left(\frac{1}{4}\right)a_{1}\left(R\right)+\left(\frac{1}{2}\right)a_{2}\left(RS\right)+\left(\frac{1}{4}\right)a_{3}\left(S\right), \end{array}$$

where *x* is the expected response of *F*_2_ for a particular dose and *a*_1_, *a*_2_, and *a*_3_ are the observed responses of the resistant (*R*), the hybrid (*RS*), and susceptible (*S*) populations to that dose.

**Figure 3:**
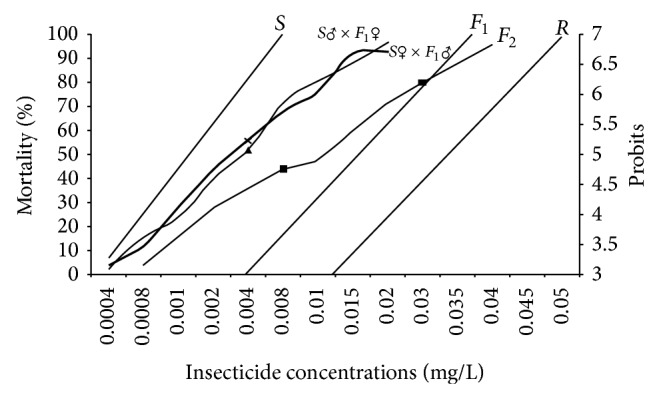
Dosage-mortality relationships of the TR and TS strains of* Aedes aegypti* showing break/inflection points on *d*-*m* lines of *F*_2_ and backcrosses. The *F*_2_ line showed two inflection points at 0.008 mg/L and 0.03 mg/L, indicating the cessation of *S* individuals and *RS* individuals, respectively. Further the backcross lines also showed one inflection point each at 0.004 mg/L indicating complete mortality of *S* individuals leaving behind only *RS* individuals signifying monogenic inheritance.

In the *F*_2_ generation, the expected outcome is 50% susceptible/resistance. The observed resistance in both the *F*_2_ crosses (9 and 10) displayed slight deviations from the expected 50%, with no significance at *P* < 0.05. The “resistance” trait can be termed monogenic if the *d*-*m* line of *F*_2_ or the backcross exhibits a distinct horizontal field of mortality across the increasing magnitude of doses. Two marked inflections in the *d*-*m* lines dividing the *F*_2_ curve at point at 0.008 mg/L indicate the mortality of the homozygous susceptible individuals (*S*) and the second inflection point at 0.03 mg/L indicates the complete mortality of the heterozygous individuals (*RS*). In addition, one inflection point was observed at 0.004 mg/L in backcross, indicating the complete elimination of *S* individuals leaving behind *RS* individuals (Figure 3).

## 4. Discussion

The present investigation reveals the mode of inheritance of temephos resistance in* Ae. aegypti*. The *d*-*m* responses of the parental strains were characterized by straight lines, indicating the homogenous nature for resistance and susceptibility. The *F*_1_ offspring also displayed a straight *d*-*m* line, confirming homozygosity of the resistance and susceptible genes involved [38]. The *d*-*m* line for *F*_1_ was found in the middle of that of the resistant and susceptible strains clearly demonstrating a heterozygous nature of temephos resistance in *F*_1_. Additionally, the calculated value for the degree of dominance (*D*) also suggests an incompletely dominant pattern of inheritance for resistance to temephos. The response of *F*_1_ hybrids to the diagnostic concentration was similar in both the crosses, which indicated that a single gene was responsible for conferring resistance to temephos. The observed *F*_2_ mortality closely approximated that expected from a 1 : 2 : 1 ratio of the *S* : *RS* : *R* types. Also, the backcrosses (*F*_1_ × *S*) were found to be almost in a ratio 1 : 1 of resistant to susceptible individuals. Thus, via both these crosses, the trait of temephos resistance appears to follow a monogenic pattern of inheritance. It is perhaps noteworthy that prior studies on* Anopheles stephensi* have reported similar genetic patterns of inheritance to several classes of insecticides [39, 40]. Resistance controlled by a single gene develops and spreads much more rapidly when compared to polygenic resistance [41, 42]. The resistance rapidly extends to new areas through migration of the resistant insect [43]. Studies on the nature of insecticide resistance have shown that the phenomenon is due to preadaptations which usually involve single gene alleles and that the emergence of insecticide resistant strains is thus a consequence of Darwinian selection [44].

The results obtained from the present work could improve our understanding of the rate of resistance development and mode of inheritance of the temephos resistance gene involved. The evidence for the simple Mendelian pattern of inheritance is based on log-dosage probit curves and agreement of the observed responses to those that may be expected in the case of monofactorial inheritance. Understanding of resistance mechanisms is crucial for developing novel strategies to circumvent and delay resistance development, controlling resistant mosquitoes, and thus ultimately bringing down the prevalence of mosquito-borne diseases. Characterizing the genes and regulatory mechanisms involved in resistance may pave the way to advanced methods of studying resistance, eventually leading to the discovery of the genes responsible for insecticide resistance [45].

Furthermore, this study can be helpful to determine any cross resistance to other groups of insecticides and understand the permanence of the resistance through succeeding generations upon removal of selection pressure. The present investigation elucidates the mode of inheritance of the temephos resistance gene in the said species, which is an excellent genetic marker for* Ae. aegypti*. Such genes are extremely useful in conducting basic and applied genetic research such as synthesis of genetic sexing strains as conditional lethal for the preferential exclusion of females during early developmental stages [46]. Moreover the characterization of single gene, which can be located on the chromosome by discovering linkage with mutant markers and which can be associated with specific detoxification enzyme, allows understanding of the resistance situation in the field and points the way to countermeasures based on remedial insecticides.

In conclusion, the ratios 1 : 2 : 1 and 1 : 1, along with the conspicuous inflections in the *d*-*m* lines, in the *F*_2_ of the *R* × *S* cross and the progeny of the backcross, provide evidence for the fact that inheritance of resistance to temephos in* Ae. aegypti* is monofactorial. Moreover the value of the degree of dominance (*D*) and the location of *F*_1_ line with respect to that of the resistant line suggest that the mode of inheritance of temephos resistance in the said species is monogenic and incompletely dominant in nature.

## Figures and Tables

**Table 1:** Inheritance pattern of temephos resistance in *Aedes  aegypti*.

S. number	Genetic crosses	Number of females tested	Number of larvae tested^**^	Resistant		Susceptible		*χ* ^2^
				Alive	%	Dead	%	
	Parental							
1	*S*♂ × *S*♀	25	1824	—	—	1824	100	—
2	*R*♂ × *R*♀	25	1707	1707	100	—	—	—

	*F*_1_ generation							
3	*S*♂ × *R*♀	25	1744	992	56.88	752	43.11	0.94^*^
4	*R*♂ × *S*♀	25	1697	1014	59.75	683	40.24	1.90^*^

	Backcrosses							
5	*S*♂ × *F*_1_♀ (cross 3)	25	1762	968	54.93	794	45.06	0.48^*^
6	*S*♀ × *F*_1_♂ (cross 3)	25	1809	1032	57.04	777	42.95	0.99^*^
7	*S*♂ × *F*_1_♀ (cross 4)	25	1794	1022	56.96	772	43.03	0.97^*^
8	*S*♀ × *F*_1_♂ (cross 4)	25	1846	996	53.95	850	46.04	0.31^*^

	*F*_2_ generation							
9	*F*_1_♂ × *F*_1_♀ (cross 3)	25	1872	1038	55.44	834	44.55	0.59^*^
10	*F*_1_♂ × *F*_1_♀ (cross 4)	25	1832	1097	59.71	730	40.28	1.88^*^

*R*: resistant; *S*: susceptible. ^*^Nonsignificant (*P* > 0.05).

^**^Late third instar larvae exposed to 0.02 mg/L for 24 h.

The expected percent mortality for cross 1 is 100, cross 2 is zero, and crosses 3–10 is 50%.
